# Weighted Domain Adaptation Using the Graph-Structured Dataset Representation for Machinery Fault Diagnosis under Varying Operating Conditions

**DOI:** 10.3390/s24010188

**Published:** 2023-12-28

**Authors:** Junhyuk Choi, Dohyeon Kong, Hyunbo Cho

**Affiliations:** Department of Industrial and Management Engineering, Pohang University of Science and Technology, Pohang 37673, Republic of Korea; cjh0102@postech.ac.kr (J.C.); dohyeono@postech.ac.kr (D.K.)

**Keywords:** machinery fault diagnosis, data analytics, domain adaptation, dataset representation, varying operating condition, unseen domain

## Abstract

Data-driven fault diagnosis has received significant attention in the era of big data. Most data-driven methods have been developed under the assumption that both training and test data come from identical data distributions. However, in real-world industrial scenarios, data distribution often changes due to varying operating conditions, leading to a degradation of diagnostic performance. Although several domain adaptation methods have shown their feasibility, existing methods have overlooked metadata from the manufacturing process and treated all domains uniformly. To address these limitations, this article proposes a weighted domain adaptation method using a graph-structured dataset representation. Our framework involves encoding a collection of datasets into the proposed graph structure, which captures relations between datasets based on metadata and raw data simultaneously. Then, transferability scores of candidate source datasets for a target are estimated using the constructed graph and a graph embedding model. Finally, the fault diagnosis model is established with a voting ensemble of the base classifiers trained on candidate source datasets and their estimated transferability scores. For validation, two case studies on rotor machinery, specifically tool wear and bearing fault detection, were conducted. The experimental results demonstrate the effectiveness and superiority of the proposed method over other existing domain adaptation methods.

## 1. Introduction

Fault diagnosis of rotor machinery is a significant task for improving process efficiency and reducing machine downtime in the manufacturing industry [1,2]. Particularly, the high cost of machinery necessitates intelligent fault diagnosis to ensure the expected functionality and performance throughout its lifespan [3]. Consequently, research on this topic has rapidly grown in recent years, paralleling the advancements of industrial machinery in terms of scale and complexity.

With the rapid progress of sensor technology and monitoring equipment, various data types can be collected during the manufacturing process. Based on this, the recent research on machinery fault diagnosis has mainly focused on data-driven methods, which can more effectively extract the crucial features of machinery faults [4]. Machine-learning methods such as support vector regression (SVR) and random forests (RFs) have shown successful performances in tool wear detection [5,6]. Lately, deep-learning methods such as auto-encoder and long short-term memory (LSTM) have outperformed machine-learning methods because they can capture complex features from a huge amount of sensor data [7].

While researchers have achieved considerable success in data-driven fault diagnosis, these methods require that the training data and the test data come from the same probability distribution [8,9]. Unfortunately, this requirement is not usually guaranteed in real-world industrial applications. For instance, machining processes under different operational conditions, such as the feed rate and the cutting depth, will not meet this requirement [10]. Since these operational parameters (e.g., cutting speed, feed rate, and rotor speed) determine data distributions of machinery data, a diagnostic model trained only on source data often performs poorly on the target domain.

Because of this issue, abundant training and test datasets must be collected under each separate operating condition to train a favorable model. However, because of the data collection costs, it is unrealistic to collect such large datasets [11] simultaneously for training the data-driven model. In scenarios where the machinery operates under a new and unseen operating condition, fault data are not available before a fault occurs. Consequently, the performance of conventional data-driven methods may be degraded when dealing with insufficient data about frequently changing operational conditions.

Various domain adaptation (DA) methods have demonstrated their efficacy in addressing the challenges posed by varying operating conditions in fault diagnosis. The DA method aims to transfer knowledge from a source domain (i.e., a label-sufficient dataset) into a target domain (i.e., a label-insufficient dataset) by exploring a domain-invariant feature space or reweighting datasets. It focuses on minimizing the data distribution discrepancy between the source and target domain so that a model trained on the source dataset can generalize better to the target dataset. DA has also been widely adopted by the manufacturing industry, particularly for fault diagnosis under varying operating conditions. 

These DA methods for fault diagnosis have shown their feasibility in dealing with varying operating conditions. However, existing DA methods tend to treat all domains equally and align them all perfectly [12]. Several works [13,14,15] attempted to demonstrate that the domain weighting schemes used on different source domains could enhance the generalizing ability to the target domain and ensemble models can handle the weights dynamically for the target [16,17]. Nevertheless, these methods typically overlook the inherent topological structures among different domains, focusing solely on one-to-one domain similarity.

Moreover, most existing DA methods primarily concentrate on quantifying data distribution distances between two domains, disregarding the valuable metadata related to manufacturing operations, resulting in a training procedure that lacks consideration for the characteristics of manufacturing processes. In contrast to fields like computer vision or natural language processing, industrial machinery operates under the control of automated control systems or operators. Consequently, the setting values of operating parameters are typically managed and stored as metadata with the datasets. Although recent studies [12,18] have shown that leveraging the metadata could improve the domain adaptation, this approach remains relatively unexplored in the field of fault diagnosis.

To deal with the aforementioned challenges, a weighted domain adaptation using a graph-structured dataset representation is proposed. The graph-structured dataset representation contains a new domain feature that considers both metadata and collected data to represent the characteristics of the manufacturing process. Then, we estimate the transferability of candidate source datasets using the constructed graph, its topological structure, and graph embedding model. Estimated weights for the target domain are then allocated based on this transferability estimation, and these weights are employed to establish a voting ensemble model. The main contributions of this article are summarized as follows.

- A domain adaptation method for machinery fault diagnosis is developed to address varying operating conditions. It focuses on a more realistic scenario where fault data from the target domain (i.e., unseen operating conditions) is unavailable during the training phase, and multiple source datasets are present. Consequently, the method aims to effectively transfer knowledge from multiple sources to enhance the fault diagnosis model’s performance for the target domain through the weighting of candidate source datasets.
- We propose the use of a graph structure to capture intricate relations among domains in the context of fault diagnosis. Within this graph structure, we introduce a novel domain feature that simultaneously considers metadata and collected data. This comprehensive representation enhances our understanding of the varying operating conditions and their effects.
- Building upon the graph structure, we present a transferability score estimation method specifically designed for multi-source domain adaptation in fault diagnosis under varying operating conditions. This method accurately quantifies the effectiveness of source domains for the target domain.
- The experimental results on both the tool wear detection and the bearing fault detection demonstrate the effectiveness and superiority of the proposed method over the baseline domain adaptation methods.

The remainder of the article is organized as follows. Section 2 provides a detailed description of the theoretical background related to this research. Section 3 offers a comprehensive explanation of the proposed method in detail. In Section 4, experimental results and the effectiveness of the proposed framework are discussed. Finally, Section 5 summarizes the key findings and presents further research topics with limitation analysis.

## 2. Theoretical Background

This article is related to several research areas in data-driven fault diagnosis, including domain adaptation, weighted domain adaptation, and transferability estimation. This section introduces previous literature that is relevant to this research.

### 2.1. Domain Adaptation

Domain adaptation aims to mitigate the data discrepancy problem by aligning the source and target domains (e.g., unseen operating conditions), thus enhancing the diagnosis model’s generalization ability. This approach has emerged as a promising solution to address the challenge of varying operating conditions in fault diagnosis.

Before delving into the details of DA, we introduce essential notations and definitions derived from survey works [19,20]. A domain is defined as a distribution D, represented as $D=\{X,\;P\left(X\right)\}$, where X denotes the input feature and $P\left(X\right)$ denotes a marginal probability distribution. Given a specific domain D, a task T consists of a label space Y and an objective function $f\left(.\right)$, which often be represented as a conditional probability distribution $P\left(Y|X\right)$. Domain adaptation is a subset of transfer learning settings, where the task remains constant, but the domains vary.

DA methods can be categorized based on their approach to transferring knowledge from source to target domains. A comprehensive review by Singhal et al. [19] classified DA methods into three categories: feature-based and two data-based DA methods. Feature-based DA methods aim to learn a domain-invariant feature representation by minimizing data distribution discrepancies between domains. For example, the feature extractor learns a common feature representation for both domains using distance measures like maximum mean discrepancy [21]. Ganin et al. [22] proposed the domain-adversarial neural network (DANN) that employs adversarial training to learn features that are agnostic to variations across different domains (i.e., source and target data) in the input data. Building on the DANN algorithm, Tzeng et al. [23] introduced adversarial discriminative domain adaptation (ADDA), which utilizes the DANN algorithm in a two-stage process, first training on labeled source data and then adapting the model to an unlabeled target domain through adversarial alignment of feature distributions.

In contrast to the feature-based approach, data-based methods aim to minimize specific distances between data distributions by assigning weights or selecting a subset of the source dataset for the target. Based on the distance, the model for the target domain only learns similar datasets to the target dataset. These methods determine the importance of source data for the target data based on distance metrics such as MMD or Kullback–Leibler (KL) divergence. Furthermore, Dai et al. [24] proposed TradaBoost, which is based on a reverse-boosting strategy where the importance of poorly predicted source data decreases at each boosting iteration.

While feature-based approaches, focusing on the role of trained models and their layers, have dominated the research area, recent attempts have highlighted the crucial role of the source dataset as well [25,26]. Likewise, several studies have shown that identifying source data can be as important as increasing the size of the source dataset. However, these distance metrics require labeled data for the target domain to accurately compute distances, making them challenging to implement in real industrial scenarios.

### 2.2. Transferability Score Estimation

Transferability score estimation is the task of quantifying the extent to which knowledge acquired from one task or domain can be effectively transferred to another, even when sufficient labeled data for the target domain is lacking. This concept plays a pivotal role in data-based domain adaptation, as accurate transferability estimations help establish relationships between domains and aid in the selection of suitable source datasets for a given target task. As a result, transferability estimation stands as an essential tool for weighting training datasets in domain adaptation, ultimately maximizing the accuracy of the target task [27].

The primary objective of transferability score estimation is to develop a score or metric that evaluates the effectiveness of domain adaptation methods in transferring knowledge from the source domain to the target domain, even in the absence of massive, labeled data. This evaluation enables the efficient assessment of domain adaptation algorithm performance across various source datasets prior to their execution.

Several works have shown the benefit of improving the accuracy of fault diagnosis by selecting high-transferability datasets. For instance, Bao et al. [28] introduced the H-score, a metric that measures the discriminative ability of the source model’s features for the target task by considering both intra-class variance and inter-class variances. Tran et al. [29] proposed the negative conditional entropy (NCE) score, derived from the empirical joint distribution of actual labels and predicted labels from the source model. Nguyen et al. [30] presented the log expected empirical prediction (LEEP) score, which replaces the source label with the average log likelihood generated by the pre-trained model. Extensions of the LEEP score, such as those utilizing Gaussian mixture models [31], aim to provide more accurate source labels. While NCE and LEEP scores are simple and practical, the estimation measure relies on the pre-trained model and its output. In an effort to generalize these measures, LogME [32] computes the logarithm of the maximum evidence based on extracted features without requiring the pre-trained model. These measures have demonstrated successful results in fields such as computer vision and speech recognition.

### 2.3. Domain Adaptation for Machinery Fault Diagnosis

The application of domain adaptation methods in machinery fault diagnosis has gained attention in recent years. For instance, Wen et al. [33] employed a distance measure of data distributions called maximum mean difference (MMD) to reduce the discrepancy between features from an auto-encoder model. On the other hand, X. Li et al. [34] utilized multi-kernel MMD, an advanced version of MMD, to align different multi-layer networks for bearing fault diagnosis.

Currently, the predominant approach in this research area has been feature-based methods, such as adversarial learning, which focuses on the role of the trained model and its layers. However, recent attempts have emerged, concentrating on the identification and removal of irrelevant source domains, which has been shown to enhance the domain adaptation performance [15]. For example, Mo et al. proposed a novel sparsity measure by improving the optimization process of invariant risk minimization for machinery fault diagnosis tasks [35]. Furthermore, several studies [36,37] have incorporated weighting mechanisms into adversarial learning. For instance, Han et al. [38] integrated a domain weighting mechanism into an adversarial domain adaptive network to assign weights to each sample from multiple source domains.

In these contexts, various novel transferability score estimation measures also have been proposed. Given that most industrial machinery datasets consist of time-series data while existing methods primarily focus on image or text data, adaptations are necessary. To tackle this challenge, Ye and Dai [39] incorporated dynamic time warping, which quantifies the distance between time series and the traditional transferability estimation, Jensen–Shannon (JS) distance. Bang [40] proposed a novel measure called expected knowledge gain, which calculates the relatedness between two manufacturing processes using metadata. This approach leverages descriptions of the manufacturing process and its significance to select source datasets for the target.

However, despite the successes achieved by these methods in enhancing domain adaptation under varying operating conditions, most of them have primarily focused on quantifying the distance between data distributions without fully incorporating metadata related to manufacturing operations during the training phase. Furthermore, these methods often consider only one-to-one distances and overlook the intricate relationships between datasets, including the possibility of negative transfer.

## 3. Proposed Method

**Figure 1:**
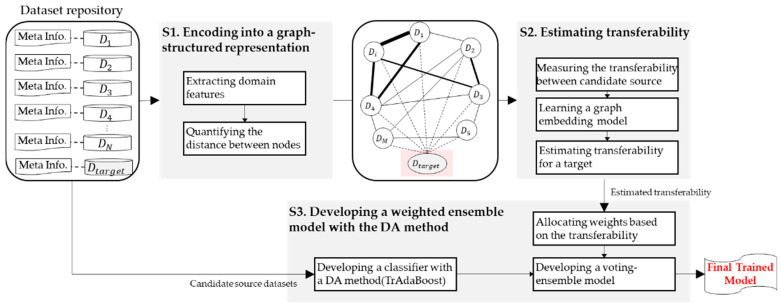
Proposed framework for fault diagnosis under varying operating conditions.

In the proposed framework (refer to Figure 1), our initial step involves converting a collection of datasets into a graph-structured representation. Then, we proceed to estimate the transferability scores of candidate source datasets by leveraging the topological structures of the constructed graph and a graph embedding model. Following the transferability estimation, we allocate weights for the target domain based on these estimates. These weights are subsequently employed in the establishment of a voting ensemble model. The details of each stage are described in the following sections.

### 3.1. Graph-Structured Dataset Representation

**Figure 2:**
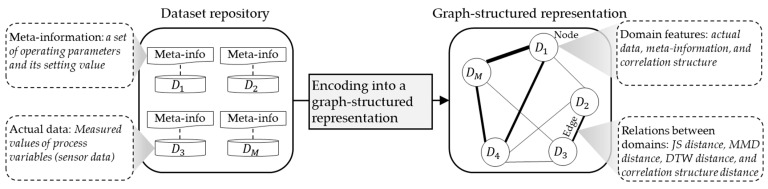
Concept of the graph-structured representation.

This stage aims to establish a unified structure that is capable of representing a collection of source datasets, capturing diverse domain features and their inter-relations. This unified structure can then be utilized to estimate transferability scores of source domains for the target domain. To achieve this unified structure, we employ a graph comprising nodes and edges. A concept of this stage named the ‘Graph-structured Dataset Representation’, is illustrated in Figure 2. In this context, each node corresponds to each domain, while an edge between two nodes refers to a relation between two different domains. The primary contribution of this article centers on the method used to define nodes and quantify edges in a manner tailored to the requirements of machinery fault diagnosis under varying operating conditions.

#### 3.1.1. Measuring Transferability Scores between Source Domains

This subsection focuses on constructing the representation for estimating transferability scores between domains. In this context, the transferability from domain $D_{p}^{S}$ to domain $D_{q}^{S}$ is defined as the effectiveness of domain $D_{p}^{S}$ in improving fault diagnostic performance under domain $D_{q}^{S}$. This is quantified by assessing the accuracy of the diagnostic model transferred from domain $D_{p}^{S}$ to domain $D_{q}^{S}$. This transferability can be quantified using the following mathematical expression:

(1) $$TF\left(p,q\right)=TF\left(D_{p}^{S},\;D_{q}^{S}\right)=Score\left(\phi \left(D_{p}^{S}\right),D_{q}^{S}\right)$$

Here, $\phi \left(D_{p}^{S}\right)$ represents a model trained on the dataset collected under domain P. $Score\left(\phi \left(D_{p}^{S}\right),D_{q}^{S}\right)$ denotes the performance score of the trained model $\phi \left(D_{p}^{S}\right)$ on dataset $D_{q}^{S}$. This formula aims to quantify the effectiveness of domain $D_{p}^{S}$ in achieving the objective related to domain Q.

Initially, the actual transferability scores between source datasets, which include labeled fault data, are assessed. This process involves training a fault detection model using a commonly employed classification algorithm, the random forest algorithm. Subsequently, the model’s performance is evaluated using an accuracy score.

Understanding the relations of transferability scores between source domains will be helpful in allocating the weight of the corresponding source domains for the target domain. Consequently, the measured transferability scores between source domains play a crucial role in constructing the graph structure.

#### 3.1.2. Extracting Domain Features for Nodes

First, we will introduce the method for characterizing features within each domain that can be effectively used to quantify the transferability between different domains. In the context of the multi-source DA setting, where a combination of datasets can compose a source dataset for the target, candidate source datasets encompass all possible combinations of source datasets. Consequently, a combination of source datasets is encoded as a node in the graph. For example, with seven source datasets originating from seven distinct domains, the resulting graph comprises 127 nodes (computed as $2^{7}-1$). Subsequently, domain features will be extracted from each of these nodes.

The focus here lies in the identification of a new domain feature that exhibits effectiveness in DA for fault diagnosis under varying operating conditions. Variations in machinery operating conditions significantly influence the correlations between process variables. For instance, in rotor machinery, higher motor speeds can lead to increased vibrations, thus altering the correlation between vibration and acceleration. These fluctuations in correlation become crucial factors contributing to the degradation of diagnostic model performance when operating conditions change. Therefore, each domain is characterized by the associations between operating parameters and process variables. The distance between different associations within a domain constitutes the elements of the edge features.

As a result, we propose the correlation structure as a novel domain feature for each node. The correlation structure encompasses associations between operating parameters from metadata and process variables from collected data. The extraction of these associations involves the computation of the Kendall rank correlation coefficient from [41], which is a statistical measure used to assess the strength of association between two sets of data. This non-parametric measure evaluates the similarity in ranking or the concordance of the order of observations between two variables without regard to the magnitude of difference between the variables. The formula for the correlation coefficient for a set of *n* paired observations (${PV}_{1},\;{PV}_{2})$ is calculated as follows:

(2) $$Coff({PV}_{1},{PV}_{2})=\frac{Number\;of\;concordant\;pairs-Number\;of\;discordant\;pairs}{Total\;number\;of\;pairs\left(n\times \frac{n-1}{2}\right)}$$

Here, concordant pairs refer to pairs where the order of variables is the same, signifying a consistent ranking. On the other hand, discordant pairs are those where the order of variables differs, indicating an inconsistent ranking. It is worth noting that tied observations, where two pairs have identical values, are considered in the calculation of the Kendall rank correlation coefficient. Each computed correlation coefficient contributes to the construction of a comprehensive correlation structure, which is typically represented as a matrix.

**Figure 3:**
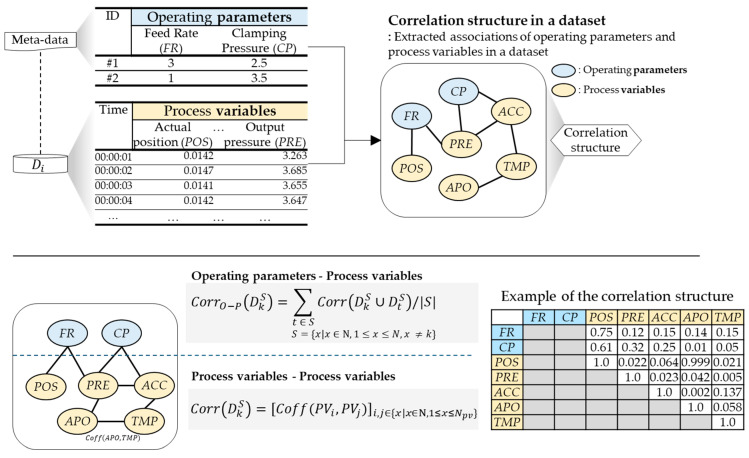
The concept and example of the correlation structure.

To refine this matrix, it was determined that correlations with an absolute correlation coefficient less than 0.5 held insignificant correlation, and the corresponding values in the matrix were replaced with 0. An illustrative example of the correlation structure is presented in Figure 3.

Moreover, we incorporated the use of raw data collected from each domain as an additional domain feature. By calculating various distances between these raw data, we quantified the relationships between domains.

#### 3.1.3. Constructing an Adjacency Matrix for Edges

**Figure 4:**
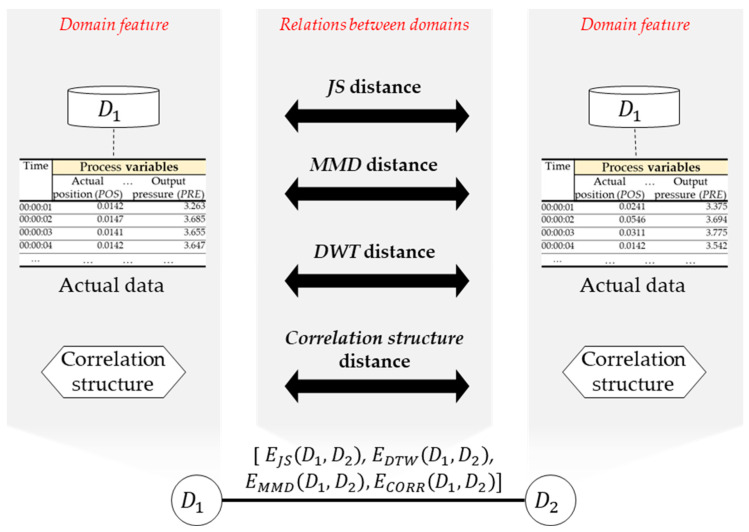
Four attributes of an edge feature.

This subsection aims to generate a matrix that contains distances between each pair of distinct nodes (i.e., domains) based on the domain features. These distances serve as the basis for connecting two nodes through an edge. Each such edge is represented as a multi-dimensional vector that signifies the relationships between the two nodes (refer to Figure 4). Typically, these relationships rely on distance measures between data distributions obtained from collected data. Examples of such measures include information divergence, MMD, JS divergence, and optimal transport (OT) [42]. In this study, we opted for JS divergence and MMD as our data-distribution similarity metric due to their ease of computation and wide applicability. However, since JS divergence does not account for temporal dependencies in time series data, we additionally incorporated dynamic time warping (DTW) as a distance measure for pairs of time series datasets. The specifics of this distance measure are expounded upon in the following phases.

JS distance $E_{JS}(S,T)$ between two data distributions (*S* and *T*) is a commonly used metric for quantifying dissimilarity. This distance measure is based on *KL* divergence, a fundamental concept in information theory. *KL* divergence quantifies the additional information required to encode data from distribution S using a code optimized for distribution *T*, as opposed to encoding it with a code optimized for distribution S itself. The *JS* distance combines *KL* divergence with an averaging step to ensure symmetry and measure overall dissimilarity in a balanced manner. Mathematically, *JS* distance can be calculated using the following expression:

(3) $$KL(S||T)=\sum [S\left(x\right)*loglog\;\left(S(x)/T(x)\right)]\;$$

(4) $$E_{JS}(S,T)=(KL(M||T)+KL(M||S))/2$$

*MMD* distance $E_{MMD}\left(S,T\right)$ is a statistical measure used to quantify the dissimilarity between probability distributions. It is commonly employed in various machine learning and statistical tasks, including domain adaptation and kernel methods. The mathematical expression for the *MMD* distance between two distributions, *S* and *Q,* is as follows:

(5) $$E_{MMD}\left(S,T\right)=||{\frac{1}{n}\sum_{i=1}^{n}\emptyset (x_{i})-\frac{1}{m}\sum_{j=1}^{m}\emptyset \left(y_{i}\right)||}_{H}^{2}$$

In the equation, n and m are the respective numbers of samples drawn from each distribution. $x_{i}$ and $y_{i}$ are the individual data points sampled from the distributions. ∅() is a feature map that transforms data into a higher-dimensional space. ||.|| represents the norm in the reproducing kernel Hilbert space.

*DTW* distance is a distance measure used to quantify the dissimilarity between pairs of time series data. Time series data frequently exhibit variations in length, posing a unique challenge when measuring dissimilarity between such datasets. To address these challenges, Sakoe and Chiba [43] introduced the *DTW* distance, which calculates the distance by optimally matching similar data points between time series. To apply the measure in the dataset similarity, each signal ${\left\{s_{i}\right\}}_{i=1}^{N}$ in datasets is divided into an equal number K of segments, each with varying lengths denoted as ${Window}_{q}^{S}={\left\{s_{i}\right\}}_{i=q}^{j+\mathrm{ceil}\;(N/K)}$. Then, we calculate DTW distances between time series windows in domain S and domain T. Subsequently, we calculate DTW distances between time series windows in domain S and domain T. The final DTW distance between the two domains is computed using the following mathematical expression:

(6) $$E_{DTW}\left(S,T\right)=\frac{\sum \sum DTW\left({Window}_{q}^{S},{Window}_{r}^{T}\right)}{K\times K}\;$$

The time complexity of calculating DTW distance between two time series depends on several factors, including the lengths of the time series and the specific algorithmic optimizations used. In its basic form, DTW has a time complexity of $O(N^{2})$, where N is the length of the time series. This quadratic time complexity arises because, in the worst case, every point in one time series is compared with every point in the other time series. In situations where time consumption is high due to a long length of time series, several approximate or fast DTW algorithms can be applied to reduce the time complexity to linear or near-linear time, O(N).

To measure the distance $E_{CORR}\left(S,T\right)$ between two correlation structures, we employed cosine similarity between the correlation structures of different domains. This similarity metric quantifies the cosine of the angle between two matrices treated as vectors. The calculation for the correlation structure distance can be expressed mathematically as follows:

(7) $$E_{CORR}\left(S,T\right)=S\times T/\;(||S||\times ||T||)\;$$

#### 3.1.4. Constructing a Graph-Structured Datasets Representation

In this subsection, a graph structure is proposed based on the extracted domain features and quantified relations between domains. Since a graph is composed of nodes and edges, its structure varies depending on how these nodes and edges are defined. In this research, two different graph structures are proposed.

**Figure 5:**
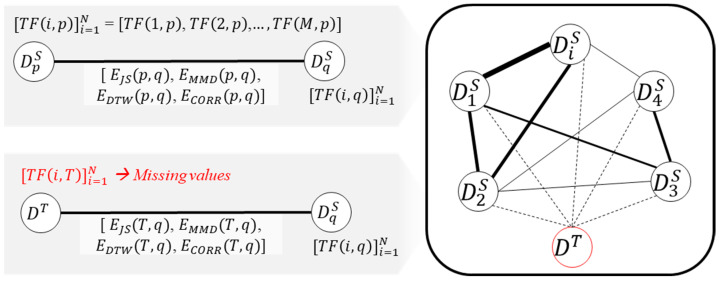
An example of the constructed graph.

In this graph structure, measured transferability scores are converted into node features (refer to Figure 5). Specifically, for the node of $D_{p}^{S}$, there are a total of N measured transferability scores between other nodes. The array of *N* measured transferability scores is converted into a node feature of the node representing $D_{p}^{S}$. Edge features consist of a multi-dimensional vector signifying the four quantified relations between the two nodes. The concept of the graph structure is illustrated in the figure. However, in the case of the target domain, the node feature of the target domain is missing since there are no labeled fault data during the training phase, and performance scores remain unknown. These missing values will be estimated in the next subsection.

### 3.2. Transferability Scores Estimation from the Constructed Graph

This subsection aims to estimate the transferability between the target domain and candidate source domains. Since there is sufficient labeled data in the target domain, the transferability between the target domain and other domains remains unknown in the constructed graph. This estimation leverages the constructed graph with a graph embedding model named GraphSAGE (Graph Sampling and Aggregation) proposed in [44].

The GraphSAGE model is well-suited for tasks involving graphs, especially when dealing with missing or incomplete node features. The key component of the model is the neighbor aggregation function, which operates by collecting information or attributes from these neighboring nodes and then aggregating or combining this information in some way to generate a summary or representation for the focal node. This function allows the model to capture the local neighborhood information of each node, which is then used to generate its embedding. This helps the model learn representations that encode the graph’s structure and relationships effectively.

The detailed training process for this model can be outlined as follows.

**Figure 6:**
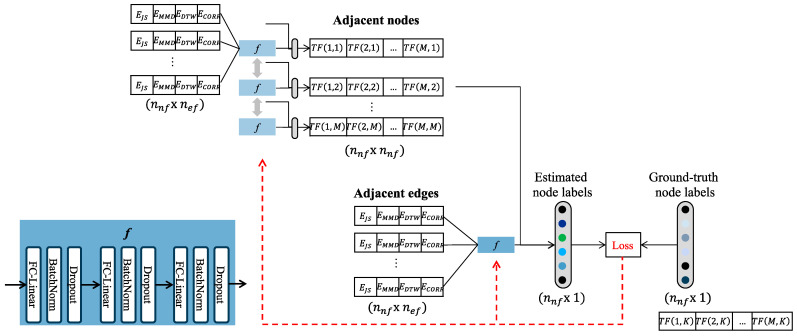
Procedure of estimating transferability scores by learning aggregation functions.

- Input preparation: The input to the model is the constructed graph itself, represented by its edge features (i.e., quantified edge features) and node features (i.e., measured transferability score). The target node with missing node features is not included in the training phase.
- Sampling neighbors: For each node in the graph, a fixed-size neighborhood is sampled. This is performed to efficiently handle graphs with nodes that have varying degrees of connectivity.
- Aggregating features: The key idea of the model is to learn a function that aggregates features from a node’s local neighborhood. Several aggregation functions can be used, such as mean, LSTM, pooling, etc. This function is learned during the training process. In this article, fully connected neural networks are leveraged for the aggregation functions (refer to Figure 6).
- Generating embeddings: The aggregated features are then combined with the features of adjacent nodes to generate the target node’s embedding. The dimension of this embedding is designed to be identical to the node feature.
- Loss calculation: The loss is calculated based on a generated embedding and a ground-truth label of the node. Specifically, a mean-squared error function is leveraged for the loss calculation.
- Backpropagation and optimization: The computed loss is backpropagated through the network, leading to the adjustment of parameters in the aggregating function using optimization techniques such as the Adam optimizer. Training occurs over multiple iterations (epochs), with each iteration dedicated to enhancing the model’s ability to minimize the loss.

After training, the trained model estimates the missing node values of the target node by using existing graph features and edge features between the target node and others. The model will leverage the learned node representations, which have been enriched by the edge features, to make these predictions.

The performance of the graph embedding model is influenced by two critical parameters: the number of neighbors selected for aggregation and the depth and structure of the aggregation process. Consequently, it is imperative to optimize these parameters, potentially employing techniques like grid search.

Furthermore, it is important to emphasize that these parameters are intricately linked to the overall time complexity of the model. Additionally, the size of the graph is the main factor of the time complexity. Therefore, it is also important to select the appropriate number of neighbors, a choice that should be tailored to the specific size and characteristics of the graph. Alternatively, exploring optimization techniques, such as graph partitioning, holds the potential to alleviate the computational burden associated with the practical implementation of GraphSAGE.

### 3.3. Two-Stage Weighting Strategy for Domain Adaptation

This subsection is focused on the development of a voting ensemble model using candidate source datasets and their corresponding estimated transferability for the target domain. Instead of constructing a single model, our approach involves establishing a voting ensemble model that combines the results of multiple models based on their assigned weights. 

Here is a step-by-step breakdown of the process.

- Developing base classifiers with instance weighting: We start by generating a diverse pool of trained models using the candidate source datasets. This involves developing individual fault diagnostic models, each trained on a specific candidate source dataset. To build these models, we employ a classification algorithm combined with a domain adaptation method to ensure the robustness of each individual model in the ensemble. Specifically, we leverage the random forest algorithm as the classification algorithm and TrAdaBoost [27] as an instance weighting mechanism. The detailed process is explained in Algorithm 1.

**Algorithm 1** Procedure of developing base classifiers with the instance weighting**Input**: A source dataset $D_{i}^{S}={\left[\left(x_{k},y_{k}\right)\right]}_{k=1}^{N_{i}}$, A target dataset $D^{T}={\left\{(x_{k},0)\right\}}_{k=1}^{N_{T}}$, A classification algorithm $h\left(.\right)$. For each iteration *p* (up to a pre-defined P): 1: Initialize weight uniformly. $2:\;\mathrm{Train}\;a\;\mathrm{classifier}\;h_{p}(.)$ using the current weights. 3: Calculate weighted error.$$e_{p}=\sum_{k=1}^{N_{i}+N_{T}}\left(w_{i}\right)\times I(y_{k},h_{p}(x_{k}))/\sum_{k=1}^{N_{i}+N_{T}}\left(w_{i}\right)$$$4:\;\mathrm{Compute}\;\mathrm{classifier}\;\mathrm{weight}.\;\beta_{p}=(1-e_{p})/e_{p}$ 5: Update weight - For misclassified source domain instances: $w_{k}^{T}=w_{k}^{T}\times \beta_{p}^{-1}$ - For misclassified target domain instances: $w_{k}^{S}=w_{k}^{S}\times \beta_{p}$ 6: Iterating K process **Output**$:\;\mathrm{The}\;\mathrm{final}\;\mathrm{classifier}\;{WC}_{i}$$.\;{WC}_{i}$$=\sum_{p=1}^{P}(\log\left(\beta_{p}^{-1}\right)\times h_{p}(x))$ After K iterations, the final model is a combination of the K classifiers.

- Domain weighting with allocated weights: The next step is to assign weights to each candidate source dataset for the voting ensemble model. These weights are determined based on the estimated transferability scores assigned to each candidate source dataset concerning a particular target dataset. Higher estimated transferability scores result in larger weight values. To ensure that the sum of all weights equals 1, the weights are adjusted accordingly. Additionally, a threshold (estimated transferability of 0) is introduced to refine the selection of source datasets for the ensemble, allowing only the best-performing source datasets to contribute.
- Building an ensemble model: The final predicted value of the ensemble model for a target domain is computed by multiplying each node’s final weight by its predicted values from the trained model with the domain adaptation method. These weighted predictions are then summed to obtain the ensemble’s prediction for the target domain. The detailed process is explained in Algorithm 2.

**Algorithm 2** Domain weighting with allocated weights**Input**: A set of base classifiers ${\left\{{WC}_{i}\right\}}_{i=1}^{M}$, A set of estimated transferability scores ${\left[TF\left(i,T\right)\right]}_{i=1}^{M}$. A test dataset ${\left\{x_{k}\right\}}_{k=1}^{T}$ 1: Normalize the weights so that they sum up to 1. $$W_{i}=\frac{TF\left(i,T\right)}{\sum_{k=1}^{M}TF\left(k,T\right)}$$2: Obtain predictions on kth sample by the base classifier *i*.$$y_{k}^{i}=predict({WC}_{i},x_{k})$$3: Get the predicted values by each classifier ${\left\{{TM}_{i}\right\}}_{i=1}^{M}$$${\hat{y}}_{k}=\sum_{i=1}^{M}y_{k}^{i}*W_{i}$$**Output**: Final predictions on the test dataset ${\left\{{\hat{y}}_{k}\right\}}_{k=1}^{T}.$

**Figure 7:**
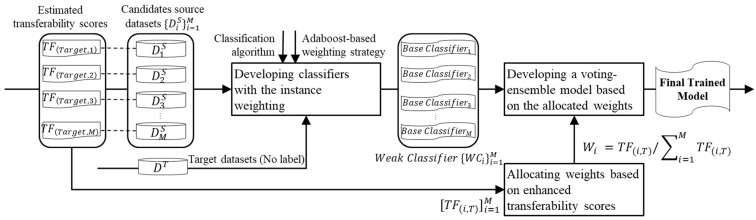
The procedure of developing an ensemble model with the DA method.

The visual representation provided in Figure 7 illustrates how the voting ensemble model operates, leveraging the weights from various source datasets to make accurate predictions for the target domain. This ensemble approach enhances the robustness and performance of the domain adaptation process in fault diagnosis under varying operating conditions.

## 4. Experimental Results and Discussion

In this section, we conduct two case studies on rotor machinery fault diagnosis under varying operating conditions to validate the effectiveness and superiority of the proposed framework. Each case study is based on real-world public datasets.

### 4.1. SMART Dataset

#### 4.1.1. Description of Dataset

**Figure 8:**
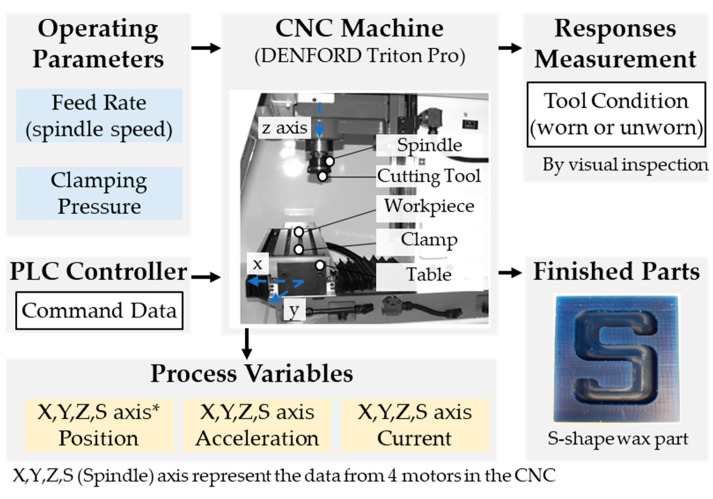
Experimental setup of a CNC milling machine in SMART Lab. (1) Adapted from “Triton user’s manual pdf” by Denford, www.denfordata.com/bb/viewforum.php?f=48, accessed on 3 April 2023; (2) Adapted from “CNC Mill Tool Wear” by Kaggle, www.kaggle.com/datasets/shasun/tool-wear-detection-in-cnc-mill, accessed on 3 April 2023.

The SMART dataset was collected from a real CNC milling machine that is part of the system-level manufacturing and automation research testbed (SMART) at the University of Michigan [45]. The testbed provided a milling machine dataset collected from a CNC milling machine under varying feed rates, clamping pressures, and tool conditions (as illustrated in Figure 8). In the CNC milling machine, feed rate refers to the relative velocity of the cutting tool along the workpiece, and clamping pressure refers to the pressure used to hold the workpiece. Depending on the characteristics of the workpiece or expected part quality, the setting values of those operating parameters may vary. In the context of this research, datasets collected under different feed rates and clamping pressures are defined as domains A to F, respectively (as detailed in Table 1).

During one such manufacturing operation, time series datasets were collected from each of the four motors (X, Y, Z, S) in the machine, where S is the spindle. A total of seven datasets were collected for each motor, resulting in a comprehensive dataset consisting of 44 variables. These datasets were sampled at a rate of 10 Hz. The time series datasets included the motor’s actual position, actual velocity, actual acceleration, command position, command velocity, command acceleration, current feedback, DC bus voltage, output current, output voltage, and output power.

#### 4.1.2. Experiment Setup

Firstly, one domain was chosen from the available seven datasets named A to G, designated as the target domain. Subsequently, all remaining datasets, excluding the chosen target, were employed as candidate source datasets for training the tool wear detection model with the DA method. Notably, we assumed that the target operating condition represented an unseen operating condition, thus restricting the involvement of solely normal (unworn tool) data from the target dataset during the training phase.

Leveraging these datasets, we proceeded to develop the tool wear detection model, employing various domain adaptation methods. Subsequently, we evaluated the model’s accuracy in predicting the target dataset. Performance assessment of the tool wear detection models was carried out employing widely recognized metrics, including accuracy scores and area under the ROC curve (AUC) scores, both of which are established for evaluating classification models.

We conducted a comparative analysis of the proposed method against several baseline methods. We began by selecting a naïve method that does not utilize any DA method and instead learns from the source dataset, assigning uniform weights to all collected data. The difference in accuracy from the “Traditional ML” serves as an indicator of the performance improvement through the application of the DA method.

- Traditional machine learning (ML): To provide a reference point, we implemented the “Traditional ML” baseline method, where equal weight was apportioned to each domain. This method exclusively relies on the source data for training the diagnostic model, devoid of any DA method.

The proposed method was also compared to the widely used DA methods from data-based and feature-based approaches. The selected feature-based baseline methods include:

- Domain-adversarial neural network (DANN) [22]: Employing adversarial training to learn features that are agnostic to variations across different domains (i.e., source and target data) in the input data.
- Adversarial discriminative domain adaptation (ADDA) [23]: Utilizing a DANN algorithm in a two-stage process by initially training on labeled source data and subsequently adapting the model to an unlabeled target domain using adversarial alignment of feature distributions.

In addition to the feature-based methods, we also considered data-based methods as baseline methods, which consist of:

- Kernel mean matching (KMM) [46]: Reweighting source data to minimize the MMD distance between domains by solving quadratic optimization problems.
- Kullback–Leibler importance estimation procedure (KLIEP) [47]: Estimating weighted importance by minimizing the KL divergence distance between domains. It assigns higher weights to the data that are more important for learning the target distribution.
- TrAdaBoost: Allocating weights based on a reverse boosting strategy where the weight of source data poorly predicted is decreased at each iteration.

To ensure a fair comparison among the above DA methods, all the methods were implemented with the same loss function (=mean squared error), learning rate (=0.001), and optimizer (=Adam). A one-class classifier, specifically the RandomForestClassifier, served as the backbone model for all DA methods. In testing, the classifier showed a 98.89% accuracy score under the traditional setting of an identical distribution, providing its capability for tool wear detection tasks. Furthermore, for methods necessitating a domain discriminator, a fully connected network structured with layers of 200-100-2 was employed. This network architecture facilitated the domain discrimination required by these specific methods in the comparative evaluation. By employing these standardized settings and model choices, the comparative evaluation of the DA methods was conducted on an equitable footing, enabling an unbiased assessment of their respective performances.

#### 4.1.3. Accuracy Comparison

In this subsection, a comprehensive comparison was conducted among six different domain adaptation (DA) methods applied specifically to the task of tool wear detection. The primary objective of this evaluation was to assess the effectiveness of each DA method in comparison to the method without any DA, serving as the baseline represented by the “Traditional ML” method. The performance improvements achieved by each DA method relative to this baseline were measured and depicted.

**Figure 9:**
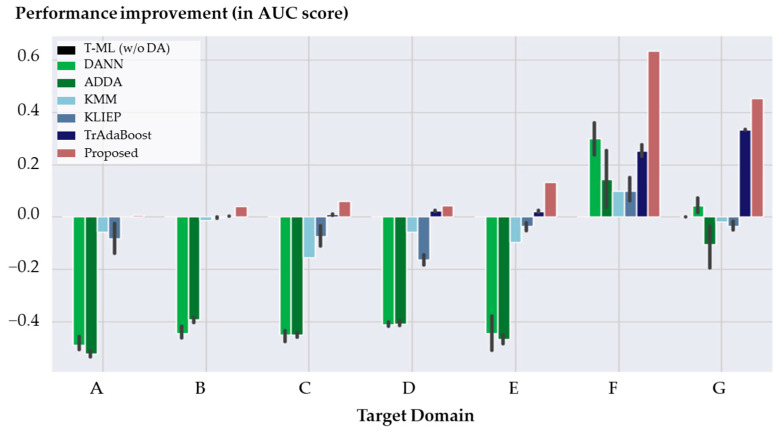
Performance comparison in the AUC score of DA methods across different target domains in tool wear detection.

Performance improvement, in this context, refers to how much the performance of a DA method is improved compared to the method without any DA. Therefore, the Y-axis of each bar graph in Figure 9 represents these performance improvements, where the values for the “Traditional ML” method are all zero, as it serves as the baseline. The X-axis represents the various target domains. For a more comprehensive breakdown of the results, Table 2 offers a detailed account of AUC scores, while Table 3 provides an in-depth analysis of accuracy scores. These tables provide a comprehensive evaluation of the performance metrics, augmenting the insights garnered from Figure 9.

The experimental results, as shown in Figure 9, indicate that the proposed method outperformed the other DA methods, achieving the highest area under the ROC curve (AUC) scores across all target domains. Notably, in the target domains F and G, the proposed method exhibited substantial improvements in AUC scores of 0.635 and 0.454, respectively. These domains operated at a feed rate of 20 mm/s, while other domains used a feed rate of 3 or 6 mm/s. The change in feed rate significantly impacted the performance in these two domains, but the proposed method effectively mitigated this discrepancy by allocating weights to each candidate source dataset.

In contrast to the other domains, the performance improvement in domain A is not statistically significant. This is likely due to the fact that the traditional machine learning method achieved an AUC score of 0.991 when applied to domain A. This suggests that, in the context of domain A, a detection model with sufficient performance can be learned even by utilizing existing data without employing DA methods.

While the TrAdaBoost method also showed performance improvements in all target domains, the observed results were inferior to those of the proposed method. This suggests that the combination of the proposed transferability estimation with the voting ensemble approach significantly enhanced the model’s ability for domain adaptation, establishing the superiority of this approach for tool wear detection under varying operating conditions.

Conversely, the feature-based DA methods demonstrated relatively lower performance improvements and, in some cases, even performed worse than the method without DA (the “Traditional ML” method). The feature-based methods tended to underperform due to their indiscriminate training with all source datasets, allowing potentially harmful datasets to negatively influence the fault diagnosis model in most of the target domains.

### 4.2. PU Bearing Dataset

In this subsection, we aim to demonstrate the generalizability of the proposed framework to a wide range of machinery fault diagnoses. To achieve this, we validated the framework for the bearing fault diagnosis under the same experimental setup.

#### 4.2.1. Description of Dataset

**Figure 10:**
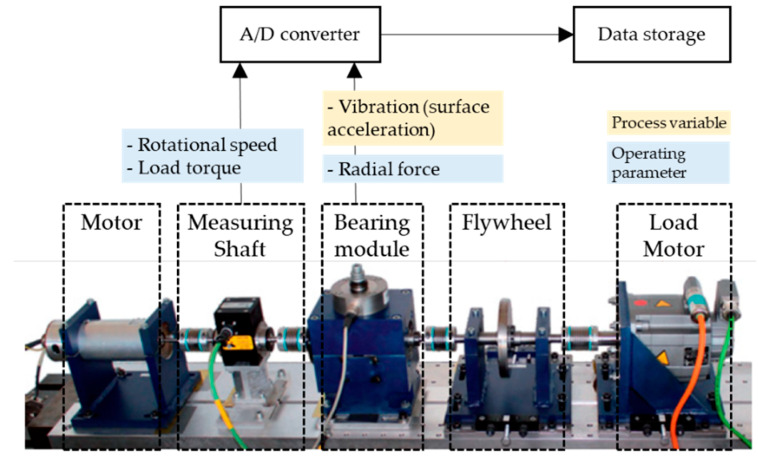
The rest rig for the PU bearing datasets.

The PU bearing datasets were collected from the test rig, which is part of the bearing data center at Paderborn University [48]. The test rig of the PU bearing datasets is depicted in Figure 10. The bearings were operated under four different operating conditions, as shown in Table 4. Each of the statuses was tested in four different operating conditions. The operating parameters were the load torque of the drive train, the rotational speed of the drive system, and the radial force on the test bearing. All three operating parameters and their setting values were constant during each measurement. During each measurement, vibration data were collected using accelerometers with a sampling frequency of 64 KHz over a duration of four seconds.

**Figure 11:**
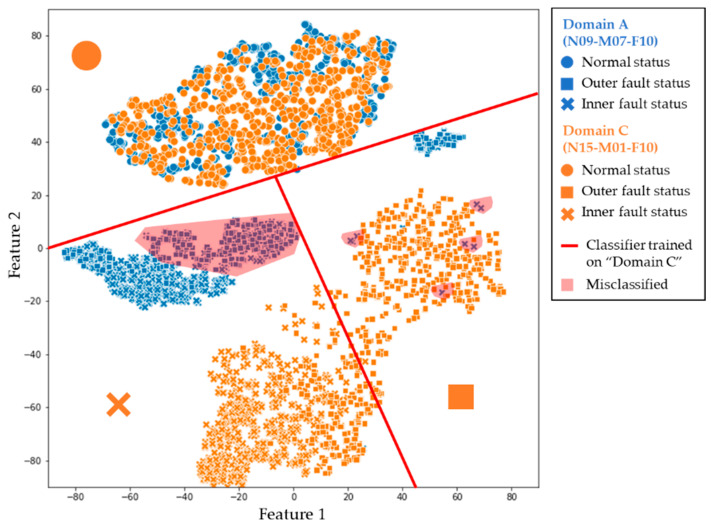
An example of the data distribution discrepancy between different domains.

Figure 11 shows the data distribution of two different domains and its negative effect on the fault diagnosis performance. The scatter plot visualizes datasets from two different domains using the t-distributed stochastic neighboring embedding (t-SNE) algorithm, a dimensionality reduction method. Instances from dataset C, collected under domain C, are represented by orange points, while those from dataset A are depicted in blue. The red lines represent the decision boundary of the classifier model that was trained from the whole dataset C and only normal data in dataset A.

Although the classifier exhibited high performance on domain C, numerous misclassified points were on domain A. This discrepancy within the PU bearing dataset domains significantly affected the models’ performance, indicating a discrepancy between the datasets across domains, thereby impeding accurate fault diagnosis.

#### 4.2.2. Experiments Setup

To implement the domain adaptation methodology, we adopted the experimental setup as outlined in [49] for the PU bearing dataset scenario. The setup involved the selection of two distinct real fault datasets, namely KA16 and KI16, as well as a normal dataset named K001. All datasets were operated under four different operating conditions denoted as domains A, B, C, and D (see Table 4). Specifically, the K001 dataset was collected under a normal bearing, the KA16 dataset was collected under the outer fault, and the KI18 was collected under the inner fault. The datasets and their corresponding operating conditions are detailed in Table 4. As a result, the task in this experiment is a multi-class (three-class) classification task under four different operating conditions. To evaluate the multi-class classification, we utilized the f-1 score instead of the AUC score. The other experimental setup was identical to Section 4.1.2.

#### 4.2.3. Accuracy Comparison

**Figure 12:**
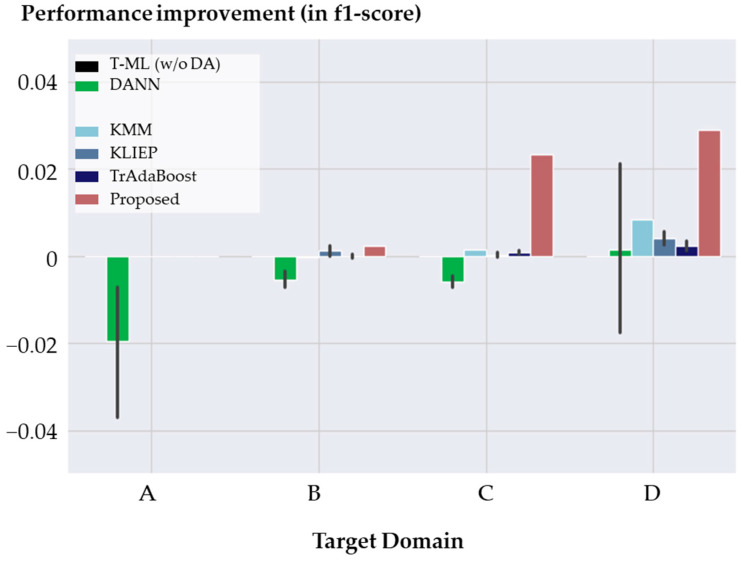
Performance comparison in the f1-score of DA methods across different target domains in bearing fault detection.

Following the completion of Section 4.1, we present the average performance improvements based on five repetitions. Similar to the previous case study, Figure 12 provides a performance comparison among various domain adaptation (DA) methods in the context of bearing fault detection across different target domains. Notably, Figure 12 excludes the results of the “ADDA” method due to its significantly lower performance, making it challenging to visualize alongside the other methods. Instead, detailed results for all DA methods are presented in Table 5 and Table 6.

As observed in the experimental results from the first case study, the proposed method consistently outperforms existing DA methods in terms of f-1 scores and accuracy scores across most target domains. This highlights the effectiveness of the proposed method not only in the previous task but also in the task of bearing fault detection.

However, it is worth noting that in the case of domain A, all DA methods failed to enhance diagnostic capability. In our experiments, the traditional machine learning (ML) method achieved f-1 scores of 0.555, 0.934, 0.941, and 0.773 for the target domains A, B, C, and D, respectively. Although the performance significantly degraded to 0.555 in domain A, it seems that the effectiveness of the proposed method was limited due to the distinct characteristics of failure data in domain A. While no performance improvement was achieved in domain A, our method showed performance improvements in other domains B to D. This demonstrated the effectiveness of the proposed method for machinery fault diagnosis under varying operating conditions.

## 5. Conclusions

This article presents a weighted domain adaptation method that leverages the graph-structured representation to achieve robust accuracies under varying operating conditions. To be specific, the collection of datasets is represented in a graph structure. Subsequently, estimated weights are assigned to the target domain based on this transferability estimation, and these weights are utilized to construct a voting ensemble model. The empirical results from two case studies in rotor machinery fault diagnosis consistently demonstrate that the proposed method outperforms existing domain adaptation techniques in terms of accuracy. These findings underscore the effectiveness of the proposed approach for fault diagnosis under diverse operating conditions.

While our research holds promise, it does come with several limitations that deserve attention. Firstly, we assume that source datasets are labeled and contain a sufficient amount of data for training diagnostic models effectively. However, in real-world scenarios, source datasets may often fall short in terms of both quantity and the availability of labeled examples. Secondly, domain adaptation may not always guarantee better accuracy by leveraging given source datasets. For example, if there is only a source dataset that is harmful to learning a diagnostic model for the target domain, the result may be poor, no matter how optimal the weight is calculated. In this case, it is necessary to ensure that the effect of domain adaptation itself will be small. Lastly, our research focuses exclusively on the classification of faults in machinery fault diagnosis.

As part of future research directions, one potential direction involves the incorporation of a measure to assess the quality of source datasets themselves when constructing the dataset graph. Such an evaluation could assist in identifying the suitability of source data for domain adaptation. To determine the potential effectiveness of domain adaptation, we suggest initializing all weights to zero when no source dataset surpasses a pre-defined criterion. Analyzing the pattern of estimated indices could provide valuable insights into the feasibility of domain adaptation in specific scenarios. Alternatively, we could explore the calculation of outliers for nodes within the expressed graph as a means to assess whether domain adaptation is likely to yield beneficial results or not. Broadening the scope of our approach to encompass regression problems could enable the development of more comprehensive and versatile fault diagnosis solutions, extending its applicability beyond classification tasks. These proposed research directions aim to address the identified limitations and further enhance the practicality and effectiveness of domain adaptation in a range of real-world scenarios and domains.

## Figures and Tables

**Table 1:** Operational conditions of each domain. Feed rate refers relative velocity of the cutting tool along the workpiece. Clamping pressure refers to pressure used to hold the workpiece in the vise.

Domain	Operating Conditions	
	Feed Rate (mm/s)	Clamping Pressure (bar)
A	6	4.0
B	3	2.5
C	3	3.0
D	3	4.0
E	6	3.0
F	20	3.0
G	20	4.0

**Table 2:** The AUC score results of tool wear detection of the proposed model and baseline models by the target domain (an average of five repetitions). The method shortcut refers to (TrAB = TrAdaBoost), (T-ML = Traditional machine learning without domain adaptation). The proposed method performs best overall.

Target Domain	Performance Improvement in AUC Score						
	T-ML (w/o DA)	DANN	ADDA	KMM	KLIEP	TrAB	Proposed
A	0.000	−0.475	−0.520	−0.057	−0.082	0.001	0.007
B	0.000	−0.433	−0.432	−0.013	−0.002	0.002	0.041
C	0.000	−0.385	−0.440	−0.155	−0.074	0.010	0.060
D	0.000	−0.413	−0.414	−0.057	−0.164	0.023	0.043
E	0.000	−0.382	−0.356	−0.096	−0.034	0.021	0.131
F	0.000	0.236	0.092	0.100	0.099	0.253	0.635
G	0.000	0.093	0.103	−0.019	−0.034	0.333	0.454

**Table 3:** The accuracy score results of tool wear detection of the proposed model and baseline models by the target domain (an average of five repetitions).

Target Domain	Performance Improvement in the Accuracy Score						
	T-ML (w/o DA)	DANN	ADDA	KMM	KLIEP	TrAB	Proposed
A	0	−0.472	−0.522	−0.058	−0.084	0.001	0.002
B	0	−0.433	−0.457	−0.010	0.000	0.002	0.002
C	0	−0.377	−0.451	−0.145	−0.070	0.011	0.018
D	0	−0.408	−0.409	−0.055	−0.162	0.023	0.030
E	0	−0.368	−0.363	−0.086	−0.034	0.023	0.093
F	0	0.053	−0.102	0.053	0.053	0.133	0.253
G	0	0.137	0.142	−0.015	−0.043	0.397	0.462

**Table 4:** Operational conditions of each domain.

Domain	Operating Conditions		
	Rotational Speed (rpm)	Load Torque (Nm)	Radial Force (N)
A	900	0.7	1000
B	1500	0.7	1000
C	1500	0.1	1000
D	1500	0.7	400

**Table 5:** The f-1 score results of the bearing fault detection of the proposed model and baseline models by experiment (an average of five repetitions).

Target Domain	Performance Improvement in the f-1 Score						
	T-ML (w/o DA)	DANN	ADDA	KMM	KLIEP	TrAB	Proposed
A	0.000	−0.020	0.029	0.000	0.000	0.000	0.000
B	0.000	−0.005	−0.263	0.000	0.001	0.000	0.002
C	0.000	−0.006	−0.323	0.002	0.000	0.001	0.023
D	0.000	0.001	0.000	0.008	0.004	0.002	0.029

**Table 6:** The accuracy score results of the bearing fault detection of the proposed model and baseline models by experiment (an average of five repetitions).

Target Domain	Performance Improvement in the ACC Score						
	T-ML (w/o DA)	DANN	ADDA	KMM	KLIEP	TrAB	Proposed
A	0.000	−0.034	−0.040	0.000	0.000	0.000	0.000
B	0.000	−0.006	−0.224	0.000	0.001	0.000	0.002
C	0.000	−0.006	−0.304	0.002	0.000	0.001	0.023
D	0.000	−0.002	0.000	0.007	0.004	0.002	0.023

## Data Availability

Data are contained within the article.

## References

[1] Li X., Liu X., Yue C., Liang S.Y., Wang L. (2022). Systematic review on tool breakage monitoring techniques in machining operations. Int. J. Mach. Tools Manuf..

[2] Fu C., Sinou J.J., Zhu W., Lu K., Yang Y. (2023). A state-of-the-art review on uncertainty analysis of rotor systems. Mech. Syst. Signal Process..

[3] Chen X., Wang S., Qiao B., Chen Q. (2018). Basic research on machinery fault diagnostics: Past, present, and future trends. Front. Mech. Eng..

[4] Wang J., Li Y., Zhao R., Gao R.X. (2020). Physics guided neural network for machining tool wear prediction. J. Manuf. Syst..

[5] Kong D., Chen Y., Li N. (2018). Gaussian process regression for tool wear prediction. Mech. Syst. Signal Process..

[6] Wu D., Jennings C., Terpenny J., Gao R.X., Kumara S. (2017). A comparative study on machine learning algorithms for smart manufacturing: Tool wear prediction using random forests. J. Manuf. Sci. Eng..

[7] Xu X., Wang J., Zhong B., Ming W., Chen M. (2021). Deep learning-based tool wear prediction and its application for machining process using multi-scale feature fusion and channel attention mechanism. Measurement.

[8] Zhang W., Deng L., Zhang L., Wu D. (2022). A survey on negative transfer. IEEE/CAA J. Autom. Sin..

[9] Li K., Chen M., Lin Y., Li Z., Jia X., Li B. (2022). A novel adversarial domain adaptation transfer learning method for tool wear state prediction. Knowl.-Based Syst..

[10] Sun C., Ma M., Zhao Z., Tian S., Yan R., Chen X. (2018). Deep transfer learning based on sparse autoencoder for remaining useful life prediction of tool in manufacturing. IEEE Trans. Ind. Inform..

[11] Xie R., Wu D. (2021). Optimal transport-based transfer learning for smart manufacturing: Tool wear prediction using out-of-domain data. Manuf. Lett..

[12] Xu Z., He H., Lee G.H., Wang Y., Wang H. (2022). Graph-relational domain adaptation. arXiv.

[13] Zhou K., Yang Y., Qiao Y., Xiang T. (2021). Domain adaptive ensemble learning. IEEE Trans. Image Process..

[14] Wei D., Han T., Chu F., Zuo M.J. (2021). Weighted domain adaptation networks for machinery fault diagnosis. Mech. Syst. Signal Process..

[15] Li Y., Dong Y., Xu M., Liu P., Wang R. (2023). Instance Weighting Based Partial Domain Adaptation for Intelligent Fault Diagnosis of Rotating Machinery. IEEE Trans. Instrum. Meas..

[16] Choi J., Lim K.J., Ji B. (2023). Robust imputation method with context-aware voting ensemble model for management of water-quality data. Water Res..

[17] Yang S., Kong X., Wang Q., Li Z., Cheng H., Yu L. (2021). A multi-source ensemble domain adaptation method for rotary machine fault diagnosis. Measurement.

[18] Chen H.Y., Chao W.L. (2021). Gradual domain adaptation without indexed intermediate domains. Adv. Neural Inf. Process. Syst..

[19] Singhal P., Walambe R., Ramanna S., Kotecha K. (2023). Domain Adaptation: Challenges, Methods, Datasets, and Applications. IEEE Access.

[20] Li W., Huang R., Li J., Liao Y., Chen Z., He G., Yan R., Gryllias K. (2022). A perspective survey on deep transfer learning for fault diagnosis in industrial scenarios: Theories, applications and challenges. Mech. Syst. Signal Process..

[21] Smola A.J., Gretton A., Borgwardt K. Maximum mean discrepancy. Proceedings of the 13th International Conference, ICONIP.

[22] Ganin Y., Ustinova E., Ajakan H., Germain P., Larochelle H., Laviolette F., Marchand M., Lempitsky V. (2016). Domain-adversarial training of neural networks. J. Mach. Learn. Res..

[23] Tzeng E., Hoffman J., Saenko K., Darrell T. Adversarial discriminative domain adaptation. Proceedings of the IEEE Conference on Computer Vision and Pattern Recognition.

[24] Dai W., Yang Q., Xue G.R., Yu Y. Boosting for transfer learning. Proceedings of the 24th International Conference on Machine Learning.

[25] Jain S., Salman H., Khaddaj A., Wong E., Park S.M., Mądry A. A data-based perspective on transfer learning. Proceedings of the IEEE/CVF Conference on Computer Vision and Pattern Recognition.

[26] Ngiam J., Peng D., Vasudevan V., Kornblith S., Le Q.V., Pang R. (2018). Domain adaptive transfer learning with specialist models. arXiv.

[27] Huang L.K., Huang J., Rong Y., Yang Q., Wei Y. Frustratingly easy transferability estimation. Proceedings of the International Conference on Machine Learning.

[28] Bao Y., Li Y., Huang S.L., Zhang L., Zheng L., Zamir A., Guibas L. An information-theoretic approach to transferability in task transfer learning. Proceedings of the 2019 IEEE International Conference on Image Processing.

[29] Tran A.T., Nguyen C.V., Hassner T. Transferability and hardness of supervised classification tasks. Proceedings of the IEEE/CVF International Conference on Computer Vision (ICCV).

[30] Nguyen C., Hassner T., Seeger M., Archambeau C. LEEP: A new measure to evaluate transferability of learned representations. Proceedings of the International Conference on Machine Learning.

[31] Li Y., Jia X., Sang R., Zhu Y., Green B., Wang L., Gong B. Ranking neural checkpoints. Proceedings of the IEEE/CVF IEEE/CVF Conference on Computer Vision and Pattern Recognition.

[32] You K., Liu Y., Wang J., Long M. LogME: Practical assessment of pre-trained models for transfer learning. Proceedings of the International Conference on Machine Learning.

[33] Wen L., Gao L., Li X. (2017). A new deep transfer learning based on sparse auto-encoder for fault diagnosis. IEEE Trans. Syst. Man Cybern. Syst..

[34] Li X., Zhang W., Ding Q., Li X. (2019). Diagnosing rotating machines with weakly supervised data using deep transfer learning. IEEE Trans. Ind. Inform..

[35] Mo Z., Zhang Z., Miao Q., Tsui K.L. (2022). Sparsity-Constrained Invariant Risk Minimization for Domain Generalization with Application to Machinery Fault Diagnosis Modeling. IEEE Trans. Cybern..

[36] Wu K., Li J., Zuo L., Lu K., Shen H.T. (2022). Weighted adversarial domain adaptation for machine remaining useful life prediction. IEEE Trans. Instrum. Meas..

[37] Qi H., Han Y., Tuo S., Zhao Q. (2023). Fault diagnosis in wind turbines based on weighted joint domain adversarial network under various working conditions. IEEE Sens. J..

[38] Han G., Xu Z., Chen C., Liu L., Zhu H. (2023). Fault diagnosis in industrial control networks using transferability-measured adversarial adaptation network. IEEE Trans. Netw. Serv. Manag..

[39] Ye R., Dai Q. (2021). Implementing transfer learning across different datasets for time series forecasting. Pattern Recogn..

[40] Bang S. (2018). Knowledge Selection and Transfer for Effective Analytics of Scarce Manufacturing Data. Ph.D. Thesis..

[41] Kendall M.G. (1938). A new measure of rank correlation. Biometrika.

[42] Peterson V., Nieto N., Wyser D., Lambercy O., Gassert R., Milone D.H., Spies R.D. (2021). Transfer learning based on optimal transport for motor imagery brain-computer interfaces. IEEE Trans. Biomed. Eng..

[43] Sakoe H., Chiba S. (1978). Dynamic programming algorithm optimization for spoken word recognition. IEEE Trans. Acoust. Speech Signal Process..

[44] Hamilton W., Ying Z., Leskovec J. (2017). Inductive Representation Learning on Large Graphs. Adv. Neural Inf. Process. Syst..

[45] Kovalenko I., Saez M., Barton K., Tilbury D. (2017). SMART: A system-level manufacturing and automation research testbed. Smart Sustain. Manuf. Syst..

[46] Gretton A., Smola A., Huang J., Schmittfull M., Borgwardt K., Schölkopf B. (2009). Covariate shift by kernel mean matching. Dataset Shift Mach. Learn..

[47] Sugiyama M., Nakajima S., Kashima H., Buenau P., Kawanabe M. (2007). Direct importance estimation with model selection and its application to covariate shift adaptation. Adv. Neural Inf. Process. Syst..

[48] Lessmeier C., Kimotho J.K., Zimmer D., Sextro W. Condition monitoring of bearing damage in electromechanical drive systems by using motor current signals of electric motors: A benchmark data set for data-driven classification. Proceedings of the PHM Society European Conference.

[49] Kim T., Chai J. (2021). Pre-processing method to improve cross-domain fault diagnosis for bearing. Sensors.

